# Hepatic vessel segmentation based on 3D swin-transformer with inductive biased multi-head self-attention

**DOI:** 10.1186/s12880-023-01045-y

**Published:** 2023-07-08

**Authors:** Mian Wu, Yinling Qian, Xiangyun Liao, Qiong Wang, Pheng-Ann Heng

**Affiliations:** 1 Guangdong Provincial Key Laboratory of Computer Vision and Virtual Reality Technology, Shenzhen Institute of Advanced Technology, Chinese Academy of Science, Shenzhen, China; 2 The Chinese University of Hong Kong, Hong Kong SAR, China

**Keywords:** Segmentation, 3D swin transformer, Multi-head self-attention

## Abstract

**Purpose:**

Segmentation of liver vessels from CT images is indispensable prior to surgical planning and aroused a broad range of interest in the medical image analysis community. Due to the complex structure and low-contrast background, automatic liver vessel segmentation remains particularly challenging. Most of the related researches adopt FCN, U-net, and V-net variants as a backbone. However, these methods mainly focus on capturing multi-scale local features which may produce misclassified voxels due to the convolutional operator’s limited locality reception field.

**Methods:**

We propose a robust end-to-end vessel segmentation network called Inductive BIased Multi-Head Attention Vessel Net(IBIMHAV-Net) by expanding swin transformer to 3D and employing an effective combination of convolution and self-attention. In practice, we introduce voxel-wise embedding rather than patch-wise embedding to locate precise liver vessel voxels and adopt multi-scale convolutional operators to gain local spatial information.

On the other hand, we propose the inductive biased multi-head self-attention which learns inductively biased relative positional embedding from initialized absolute position embedding. Based on this, we can gain more reliable queries and key matrices.

**Results:**

We conducted experiments on the 3DIRCADb dataset. The average dice and sensitivity of the four tested cases were 74.8$\%$ and 77.5$\%$, which exceed the results of existing deep learning methods and improved graph cuts method. The Branches Detected(BD)/Tree-length Detected(TD) indexes also proved the global/local feature capture ability better than other methods.

**Conclusion:**

The proposed model IBIMHAV-Net provides an automatic, accurate 3D liver vessel segmentation with an interleaved architecture that better utilizes both global and local spatial features in CT volumes. It can be further extended for other clinical data.

## Introduction

### Background

CT liver vessel segmentation is essential for 3D visualization, path planning , and guidance in interventional liver surgery [28, 29]. However, the vessel and liver backgrounds show similar intensity values on CT images due to their similarity in the enhancement characteristics. They are curvy, twist, occlude one another, and sometimes are seriously distorted by liver tumors. Due to the intensity similarity and complex structure of the liver vessel, accurate liver vessel segmentation is still challenging. Nowadays, accurate liver vessel segmentation heavily relies on doctors’ manual segmentation, which is hugely time-consuming and subject to the experience and skills of the experts [5].

Therefore, automatic vessel segmentation has triggered a broad discussion in the community. Even though some deep learning methods achieved big success on organ segmentation tasks, they cannot perform well in vessel segmentation due to the considerable variations of vessel structure and unbalance between backgrounds and vessels. Most recent work are designed based on FCN [20], U-net [26], and V-net’s [22] variants. They heavily rely on convolution layers, which integrate multi-scale local information to get passable results. Yet convolution’s limited reception field does not have long dependencies and enough global features, it can hardly accurately distinguish variant vessel margins and segment minor vessels. Therefore, developing a liver vessel segmentation method that adds long dependencies and utilizes global spatial features is necessary.

### Related work

Current liver vessel segmentation methods can be roughly classified into traditional region-based methods, edge-based segmentation methods and deep learning-based methods. As region-based methods do not perform well in vessel segmentation, we review most related work in the latter two categories. Since we use the transformer model as our backbone, we also review the newest work related to the transformer model. A more comprehensive literature survey can refer to [7].

*Traditional methods*

Edge-based methods can be further classified into image filtering and enhancement algorithms, tracking-based algorithms [23]. Filter and enhancement algorithms extract the volume with a common process called filtering to reduce the noise, then enhance the vessels by applying image gradients or multi-scale high-order deviations, particularly the second derivatives of the angiographic images to extract high-frequency information [16, 21]. Besides, Pamulapati et al. [24] introduced a vessel segmentation method based on the medial axis enhancement filter. Tracking-based algorithms focus on the predefined vessel models and track the minimum cost path. Friman et al. [9] proposed to track many hypothetical vessel trajectories at the same time, which improved the results in low contrast conditions. Cetin et al. [3], Cetin and Unal [2] presented the tubular structure segmentation method, which utilized a second-order tensor from directional intensity measurement and employed a higher-order tensor based on cylindrical flux-based to construct the vascular structure.

*Deep learning-based methods*

Most deep learning-based liver vessel segmentation work rely on CNN-based architecture, specifically, U-net [26] and its variants, as well as little attempts by FCN [20] and V-net [22]. In chronological order, early-stage vessel segmentation methods like retinal vessel segmentation are based on 2D methods. Later, with the segmentation targets changed to 3D images, 3D methods became mainstream. Fu et al. [10], Li et al. [18] have proposed the segmentation method for the retinal vessel from 2D images. These methods can handle small objects in 2D slices, however, the vessel segmentation on the liver, brain, or lung are volume tasks. Most 2D methods cannot transfer to 3D images directly due to space continuous along the Z-axis, which omits essential information. Therefore, the current state of art solutions for liver vessel segmentation focus on 2D multi-path(2.5d) and 3D methods. Kitrungrotsakul et al. [15] specifically proposed three DenseNets with the shared kernel that fit for resampling three planes(sagittal, coronal and transverse planes) patches from IRCADb dataset called 2.5D method. Çiçek et al. [6] extend UNet from 2D image to volume, which fused multi-scale 3D convolution feature called 3D-UNet. In order to employ the 3D representation of liver vessel features, Huang et al. [12] proposed the variant of 3D-Unet fit the problem worked well, and their evaluation of IRCADb incomplete annotations further improved the result. Yu et al. [33] added the residual module into the 3D-UNet that provided more residual features. Xu et al. [31] employed a 3D-FCN frame for this task. However, a reasonably supervised deep network model has to be trained on a large dataset with high-quality labels, and the current datasets cause the noise labels to hurt the model performance. Lately, Yan et al. [32] proposed a way to fuse self-attention into 3D U-net that improved segmentation details as a great attempt.

*Vision transformers and 2D swin transformer*

The self-attention mechanism allows transformers to dynamically extract the important features of word sequences and learn their long-range dependencies. This notion has recently been extended to computer vision by defining the vision transformer [8], which aims at the image recognition task. By taking 2D image patches with positional embeddings as input and pre-trained on large classical datasets, ViT achieved comparable results with the CNN-based methods. In medical image tasks, more recent methods like [4, 34] enjoyed the benefit of both CNNs and transformers. Efforts of Chen et al. [4] firstly utilize CNNs to extract low-level local features and transformers to catch global intersections. Currently, based on the shifted windows mechanism, Liu et al. [19] proposed Swin transformer that can learn hierarchical object concepts at different scales by applying appropriate downsampling to feature maps that achieved state-of-art semantic segmentation. Inspired by swin-transformer, Swin-Unet [1] firstly employed hierarchical transformer blocks with integrated encoder and decoder to build U-shape architecture. This work improved transUnet’s result on medical multi-organ segmentation tasks. For 3D segmentation, Karimi et al. [14] tentatively replaced the 3D convolutional operators with transformers as the backbone to build the model. They first split the local volume block into 3D patches and embedded them into a 1D sequence through ViT’s self-attention design. Compared to these methods, our IBIMHAV-Net inherits the advantages of convolution in encoding precise spatial information and using inductive biased self-attention in hierarchical representation that helps to overcome connectivity and variance of liver vessel segmentation.

### Proposed method

Motivated by existing 2D swin-transformer [1, 19] and past vision transformer attempts [4, 8, 11], we propose a transformer-based architecture for volumetric liver vessel segmentation which better utilize global features and long dependencies. The main advantages and contributions of the proposed method are as follows:

1. We propose a network architecture by expanding swin transformer to 3D and combining convolution and self-attention to play their strengths. For self-attention, the global spatial information has been encoded by embedding, and long dependencies have been entangled by our designed 3D transformer block. For convolution, multi-scale convolutions in the local feature path and downsampling/upsampling layers help to encode precise local information and capture hierarchical resolution features.

2. We introduce the voxel-wise rather than patch-wise embedding as the initial transformer input to fully utilize volumetric information, which transforms volumetric prediction to the sequence-to-sequence prediction in hierarchical resolution features.

3. We propose the Inductive Biased multi-head attention(IB-MSA) which changes the positional embedding way that learns biased positional embedding with initialization of absolute 1-dimensional embedding in the transformer blocks. Thus dramatically improving liver vessel segmentation results.

## Methodology

The proposed method starts with dataset preprocessing. Then we introduce the architecture of our framework, namely Inductive BIased Multi-Head Attention Vessel Net(IBIMHAV-Net), including the details of our 3D transformer design and inductive biased multi-head attention mechanism. Finally, we describe post-processing which reduces some discrete inaccurate results.

**Fig. 1:**
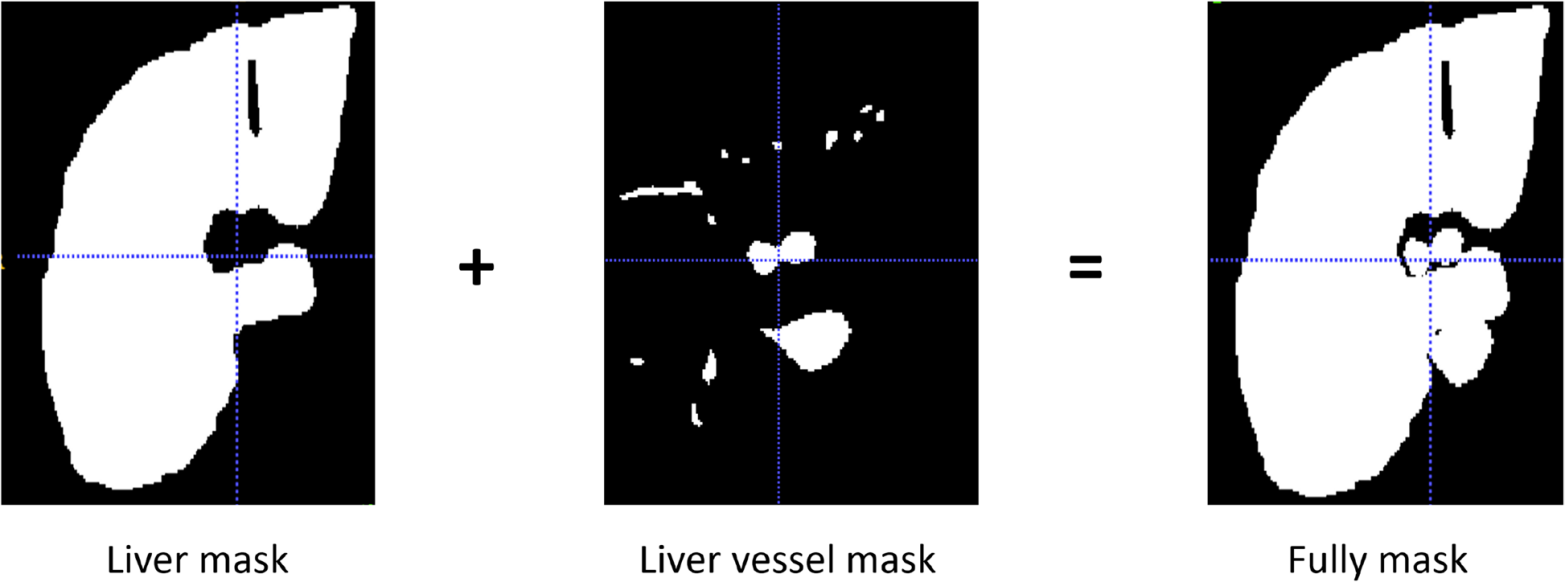
supplement of vessel mask used in the training set

**Fig. 2:**
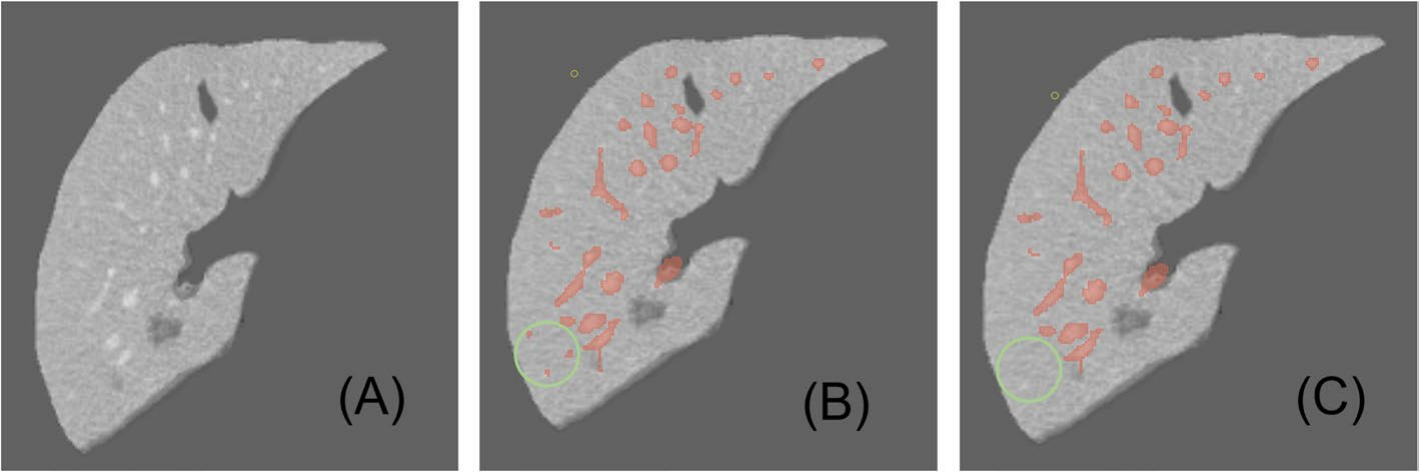
Effect of our pre-processing, (A) is the original, (B) represents before preprocessing, (C) represents the CT after pre-processing

### Preprocessing

Preprocessing plays an essential role and affects the segmentation results significantly [12, 13]. For example, applying preprocessing to lower the background noises and augment image contrast. Therefore, we arrange preprocessing as 4 steps: (1)3D IRCADb provides 20 groups of CT images, liver vessel masks, and liver masks. We crop CT images and liver vessel masks to the liver region boundary as the ROI. Then adjust to the size to 256x256x192 to unify the model input. (2)We truncate the intensity of all voxels in the volumes to the range of [-50, 250] HU to reduce the irrelevant details and increase image contrast. (3)In order to retain enough vessels’ continuity features, we add a vessel mask outside the liver as a supplement of vessel information(Eg. Fig. 1). (4)Images are normalized to zero mean and unit variance. Because most liver vessels are quite small, we keep images with their original resolution can prevent artifact errors caused by resampling Fig. 2.

**Fig. 3:**
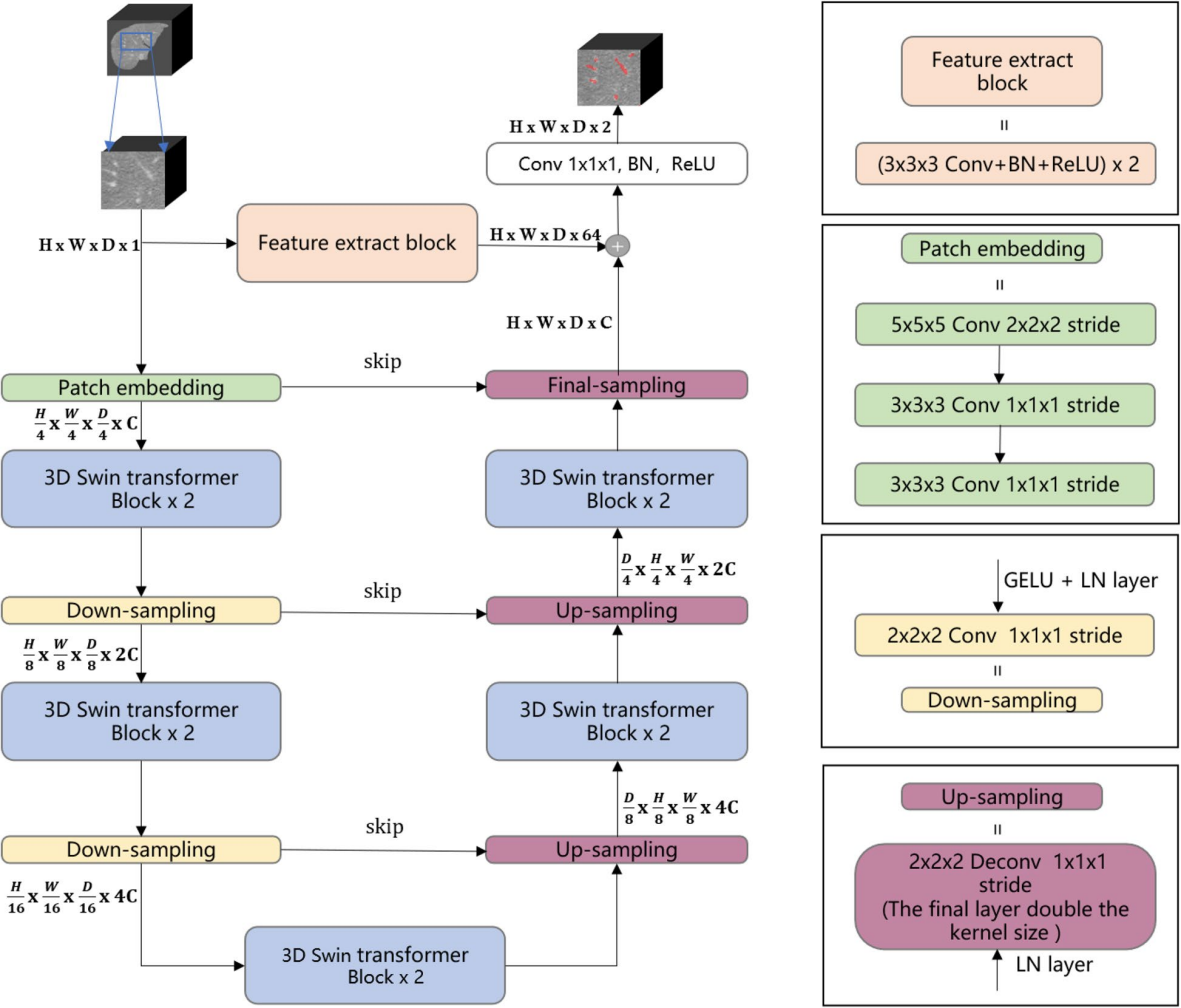
Left: Architecture of IBIMHAV-Net. Right: Compose of conv embedding, feature extraction block, up-sampling layers and down-sampling layers

### Overview of the architecture

The overall proposed architecture is shown in Fig. 3 Left, which illustrates its U-shape form which includes encoder and decoder. We introduce the U-shape end-to-end Transformer network IBIMHAV-Net, which employ pixel-wise embedding way for transformers. Our model’s long-range contextual interactions and precise spatial locate dependencies was provided by inductive biased multi-head self attention(IB-MSA) modules. The U-shape structure combined with feature extract path and three skip connections between multi-scale feature pyramids of encoder and decoder in a symmetrical manner. It helps to keep fine-grained details between transformer blocks. The feature extraction block and interleaved convolution up/downsampling layers gain accurate local spatial information and abundant local features.

### Encoder

Past vision transformer work like [4, 8, 19] have complete encoder part, yet they did not design a 3D encoder. Our architecture built up a path that includes 3D embedding block, downsampling layers combined with our transformer block design. In the encoder, the input is a 3D volume patch randomly cropped from full volume. Then we represent each 3D patch as HxWxD where H,W,D denote the height, width, and depth of each input patch, respectively. Thus, the 3D convolution embedding layer obtains tokens, with each patch/token consisting of a 128-dimensional feature. A linear embedding layer is then applied to project the features of each token to a 1D sequence length denoted by C. The outputs of the patch embedding block are connected to five 3D swin transformer blocks interspersed with down-sampling blocks.

**The patch embedding block** The linear embedding part is essential in the original swin transformer design [19], the Swin-T version first splits the one channel vessel patch into non-overlapping patches size of into 1-D sequence, then followed with big convolutional kernels in the linear embedding layer to extract small patches features. However, our task needs more precise spatial information with larger input volume. Our embedding layer first tokenized the vessel volume patch $\mathcal {X} \in \mathcal {R}^{H \times W \times D}$ into high dimensional tensor. This high-dimensional tensor represents as $\mathcal {T} \in \textrm{R}^{\frac{H}{4} \times \frac{W}{4} \times \frac{D}{4} \times C}$, where ${\frac{W}{4} \times \frac{D}{4} \times C}$ is the patch tokens and C represents the length of sequence which is 128(discussed in 3.3). Due to the variant and complex vessel structure, we design the successive large kernel convolutional combinations for pixel-wise level sequence encoding instead patch-size encoding. Moreover, this setting reduce computational complexity with same range of receptive field to accommodate long sequence. After every convolutional layer followed one GELU and one layerNorm layer to fully embedding as 1-D sequence. The kernels and strides are set as Fig. 3 Right since the input volumes were nearly squares to fit the model.

**Down-sampling layer** The swin transformer blocks used neighboring concatenate operations in past 2D tasks [1, 19]. However, we find that easy convolution with small strides worked better. It also needs a GELU layer and a Layer Norm to keep the normalization of processing measures to refine the feature map mapped to [0, 1] to keep the sensitivity of the model. It works better than Batch Normalization (BN) and ReLU activation function in our architecture.

### 3D swin transformer block with Inductive Biased MSA Module

After passing patch embedding block’s, the high dimensional sequence tensor $\mathcal {T}$ is put into transformer blocks. Compare to original Swin transformer, our method conduct self-attention in a hierarchical path and compute self-attention within 3D patches volume with bias focusing on block edge segmentation (i.e. IB-MSA, bias positional multi-head self-attention) instead 2D shift window.

**3D transformer block** In the tail of embedding block, the sequence is transformed to the high-dimensional tensor in swin transformer blocks. The main idea is to fully mix the captured long-term dependencies with hierarchical object concepts at various scales by following down-sampling convolution and global spatial information from the beginning embedding block.

In order to represent the workflow in our design, let the high-dimensional tensor $\mathcal {T} \in \mathcal {R}^{L \times C}$ reshape as $\hat{\mathcal {T}} \in \textbf{R}^{N \times P \times C}$ by passing through IB-MSA, where *N* is the number of tiny local volumes, $P = S_{H} \times S_{W} \times S_{D}$ denotes the number of patch tokens in each volume. $\left\{ S_{H}, S_{W}, S_{D}\right\}$ stand for the size of tiny local volume. To fit to our task’s various shape of vessel CT scans, this setting could cover all patch tokens of the last transformer block in the encoder. Because of different sampling quality between datasets, it may not be reasonable to brute-force pad the data in order to satisfy fixed $\left\{ S_{H}, S_{W}, S_{D}\right\}$. Therefore, the cropped patch X needs to be adaptively adjusted in order to fit the size of local volumes. And we set $\left\{ S_{H}, S_{W}, S_{D}\right\}$ on IRCADb to $\left\{ 4, 4, 4\right\}$.

Following the baseline [1], we present two successive transformer blocks. The main difference is that our computational unit is built for 3D volumes rather than 2D windows. Based on above volume partitioning way, the continuous swin transformer can be formulated as follows:

1 $$\begin{aligned} \hat{\mathcal T}^{l}=IB-M S A\left( L N\left( {\mathcal T}^{l-1}\right) \right) +{\mathcal T}^{l-1} \nonumber \\ {\mathcal T}^{l}=M L P\left( L N\left( \hat{{\mathcal T}}^{l}\right) \right) +\hat{{\mathcal T}}^{l} \nonumber \\ \hat{\mathcal T}^{l+1}=Shifted \ IB-M S A\left( L N\left( {\mathcal T}^{l}\right) \right) +{\mathcal T}^{l} \nonumber \\ {\mathcal T}^{l+1}=M L P\left( L N\left( \hat{{\mathcal T}}^{l+1}\right) \right) +\hat{{\mathcal T}}^{l+1} \end{aligned}$$

Here, *l* expresses the layer number, MLP represents multi-layer perceptron. IB-MSA is our bias multi-head attention and it has the 3D shifted version.

**Fig. 4:**
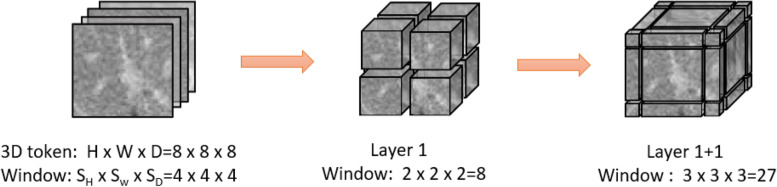
An illustrated example of $3 \textrm{D}$ shifted windows. The input size $H^{\prime } \times W^{\prime } \times D^{\prime }$ is $8 \times 8 \times 8$, and the 3D window size $M \times M \times M$ is $4 \times 4 \times 4$. As layer *l* adopts regular window partitioning, the number of windows in layer *l* is $2 \times 2 \times 2=8 .$ For layer $l+1$, as the windows are shifted by $\left( \frac{S_{H}}{2}, \frac{S_{W}}{2}, \frac{S_{D}}{2}\right) =(2,2,2)$ tokens, the number of windows becomes $3 \times 3 \times 3=27$. Though the number of windows is increased, the efficient batch computation in [19] for the shifted configuration can be followed, such that the final number of windows for computation is still 8

**The 3D shifted window partitioning** For an efficient self-attention model, we propose the method within local 3D windows. The windows are arranged to evenly partition the image in a non-overlapping style(Fig. 4 middle). Supposing each window contains MxMxM patches, we extend the naive 2D MSA(e.g. swin transformer) to 3D. The computational complexity of IB-MSA on a volume of h*w*d patches is:

2 $$\begin{aligned} \Omega (\textrm{MSA})=4 h w d C^{2}+2(h w d)^{2} C \end{aligned}$$

where the h, w, and d are fixed. However, the global self-attention computation is unaffordable in 3D successive transformer blocks. And we designed both scalable windows and tiny local self-attention to reduce a huge amount of computing resources. Firstly, we schedule local tiny patches $\left\{ S_{H}, S_{W}, S_{D}\right\}$ to introduce more interactions between local volumes and volume$\left\{ h,s,d \right\}$.

3 $$\begin{aligned} \Omega (\textrm{IB}-\textrm{MSA})=4 h w d C^{2}+2 S_{H} S_{W} S_{D} h w d C \end{aligned}$$

Besides, the shifted window layers reduced the computational complexity by the efficient batch computation shown in Fig. 4. In the next layer l+1, the shifted IB-MSA reduces computational complexity by using half compressed tiny volume $\left( \frac{S_{H}}{2}, \frac{S_{W}}{2}, \frac{S_{D}}{2}\right)$ that choose M=2 and mask out the padded values when computing attention. The self-attention computation in the new windows crosses the boundaries of the previous windows in layer l, providing connections among them shown in Fig. 4 right.**IB-MSA and relative position bias matrix** Some recent researches [1, 8, 19] have shown that there are a lot advantages in bias to self-attention computation. Here, we intuitively change the biased focus on the edge of segmentation volume by introducing 3D relative position bias $B\in \mathbb {R}^{M^{2} \times M^{2} \times M^{2}}$ for each head as:

4 $$\begin{aligned} \text{ Attention } (Q, K, V)= \text{ SoftMax } \left( Q K^{T} / \sqrt{d}+B\right) V \end{aligned}$$

where $Q, K, V\in \mathbb {R}^{ P \times d}$ are the query, key and value matrices; *d* is the dimension of query and key features, and *P* is the number of patch tokens in a $3\textrm{D}$ window. Since the relative position along each axis lies in the range of $[-2M+1, 2M-1]$, the positional mask have a big value other than B item. we parameterize a smaller-sized bias matrix $\hat{B} \in \mathbb {R}^{(2M-1) \times (2M-1) \times (2M-1)}$, and values in *B* are taken from $\hat{B}$.

Unlike standard self-attention module totally discards the position information and is perturbation equivariant which cannot model the image content in high structure, e.g. UNETR [11]. Swin transformer and swin-Unet [1, 19] use relative bias position embedding. However, original relative bias may lose some inductive bias such as locality and translation equivariance that has been mentioned in swin-transformer ablations. Moreover, the spatial invariance is crucial for our transformer interleaved with convolution design and small medical image datasets. This type of position embeddings carry no information at patches and all spatial relations between patches need be learned from zero [8].

**Fig. 5:**
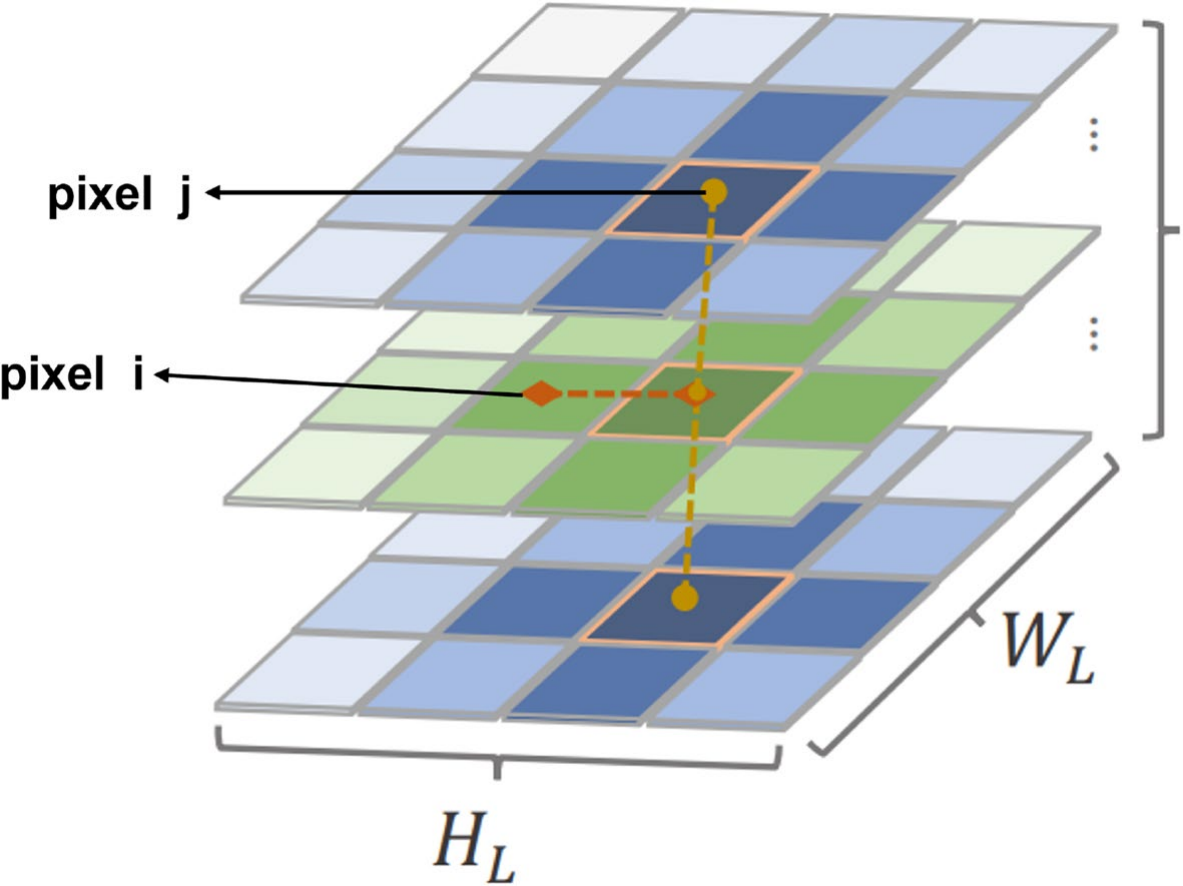
Here is the detail of mechanism in our inductive biased attention computing in swin transformer blocks

To overcome the above problem, we first initialize the pair-wise attention computing logit with 3D absolute relative bias in patch embedding and the first 3D swin-transformer block. In addition, we The pair-wise attention logit before softmax using relative position encoding between pixel $i=\left( i_{x}, i_{y}, i_{z}\right)$ and pixel $j=\left( j_{x}, j_{y}, j_{z}\right)$. where $q_{i}$ is the query vector of pixel $i, k_{i}$ is the key vector for pixel $j, r_{j_{x}-i_{x}}^{W}$ , $r_{j_{y}-i_{y}}^{H}$ $r_{j_{z}-i_{z}}^{D}$and are learnable embeddings for relative width $j_{x}-i_{x}$ and relative height and depth. Therefore, the relative position in pixel-wise attention computing and inductive bias can both be guaranteed in IB-MSA logit computing in Eq. 5 and Fig. 5.

5 $$\begin{aligned} l_{i, j}=\frac{q_{i}^{\top }}{\sqrt{d}}\left( k_{j}+r_{j_{x}-i_{x}}^{W}+r_{j_{y}-i_{y}}^{H}+r_{j_z}-i_{z}^{D}\right) \end{aligned}$$

**Fig. 6:**
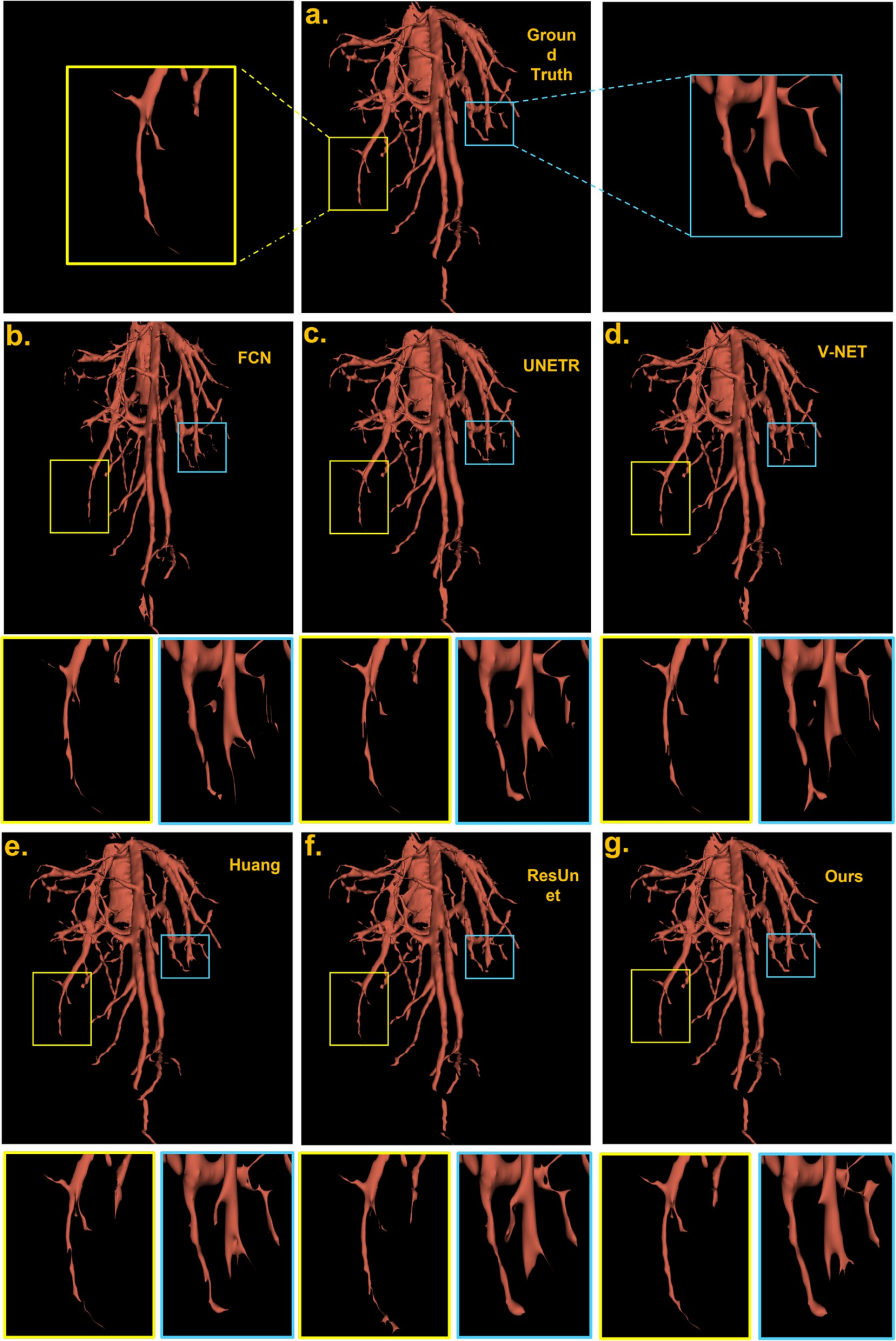
Visualization and comparison of proposed deep learning method and state-of-art machine learning-based methods using raw volume as input with post-processing. Three row indicates different genres of methods. First row: (a) ground truth result which is most similar to our result. Second row: (b), (c), (d) the traditional 3d medical image methods. Third row:(e), (f), (g) the modern deep learning methods in the journals and our method

Our specific setting improved liver vessel edge segmentation in Fig. 6 and we observe slight improvement with this bias complement with absolute position. The comparison of other methods is shown in Table 1.

**Table 1:** Precision/time trade-off

Method	Memory(MB)	Method	Memory(MB)
FCN	15.53	UNETR	92.58
V-NET	17.29	ResUnet	36.65
Huang et al.	19.36	Ours	103.5

### Decoder

In the decoder part, the transformer blocks are similar to the encoder in another direction. Moreover, the up-sampling blocks use deconvolution operator with small kernels and strides which can recover low-level features to high-resolution details quickly if it is combined with skip connections. In the final stage, the transformer result is combined with the local extraction block to output the end-to-end result.

### Weighted Loss Function

Liver vessels only exist in a small region of the liver, and unbalanced foreground(hepatic vessels) and background classes(liver) often cause predictive deviation and bias the classification to the background with more voxels. Traditional dice coefficient calculated as:

$$\begin{aligned} \text {Dice}(P, G)=\frac{|P \cap G|}{|P \cap G|+0.5(|P-G|+|G-P|)} \end{aligned}$$

where *P* is the predicted labels, *G* is the labels of the ground truth. It is hard to achieve desired segmentation results with vessels edge and small branches. The similarity matrix of dice coefficient with special penalty weight parameter as $WD(P, G, \beta )$(weighted loss) has been proposed to design loss function [12] as follows:

6 $$\begin{aligned} WD(P, G, \beta )=\frac{|P \cap G|}{|P \cap G|+0.5 \beta (|P-G|+|G-P|)} \end{aligned}$$

where $\beta$ determined the weight of the number of correctly classified foreground voxels and misclassified voxels.

Since our task has 2 class labels, we can take foreground and background as the first and second classes, respectively. Then Eq. (5) becomes:

7 $$\begin{aligned} WD(\beta )=\frac{\sum _{i=1}^{N} p_{0 i} g_{0 i}}{\sum _{i=1}^{N} p_{0 i} g_{0 i}+0.5 \beta \left( \sum _{i=1}^{N} p_{0 i} g_{1 i}+\sum _{i=1}^{N} p_{1 i} g_{0 i}\right) } \end{aligned}$$

where $p_{0 i}$ and $p_{1 i}$ are the probabilities that voxel *i* belongs to the foreground (liver vessel) and the background (liver), respectively in the softmax layer output result. $g_{0 i}$ and $g_{1 i}$ are the labels of voxel *i* in the annotated data for liver vessels or liver with values 0 or 1, respectively.

From Huang et al. [12]’s studies, the gradient of similarity in Eqs. (6) to 2 variant shows the weight of the liver(background) and liver vessel(foreground) do not need a pre-trained method unlike Chen et al. [4], which provided the initial training weights from other models or datasets. Moreover, the proposed algorithms adjust the penalty for misclassified voxels by selecting $\beta$ as 6 can both optimize the dice value and sensitivity in our model.

### Post-processing

Due to limitations of the GPU’s memory, we cannot put full-size volumes into our model. It can cause residual errors in the patch edges. Therefore, connected component analysis is performed on the vessels after being trained by IBIMHAV-Net. To remove some noises caused by classification, regions with small partitions(less than 180 m$m^3$)are removed.

## Experiments and results

### Data augmentation and experiment material

3Dircadb-01 dataset is currently available with liver and liver vessel contours suited to our training and evaluation of liver vessel segmentation algorithms. The dataset includes 20 contrast-enhanced CT volumes with various image resolutions, vessel structures, intensity distributions, and contrast between liver and liver vessels. To keep the accuracy, transform invariance, and robustness of our network, the training set and test set should have clear, abundant hepatic vessel structures with different intensity ranges, and contrast with the background and vessels. The liver vessel appearances should be similar in both training and testing datasets, so we deliver some experiments. By observing the voxel numbers and statistical data, The 3DIRCADb dataset includes 6 simple samples and 14 challenging samples. Finally, we choose 16 volumes and 4 volumes as training/test data separately (both include simple and challenging samples) based on hand selection in each experiment. For the 16 training sets, we have to apply some image amplification methods for increasing our training set. For a sample in the training case, the fixed rotation set for 60$^{\circ }$, 270$^{\circ }$ then add random translation from -25 to +25 pixels to get three times data as an augmentation strategy. In both the training and testing datasets, the original pixel spacing varied from 0.56mm to 0.87mm, slice thickness varies from 1.25mm to 2mm and slicer varied from 113 to 225.

Our proposed method was implemented using Python 3.8 and PyTorch 1.9.0. All experiments were conducted on an Nvidia A6000 GPU with 48GB memories. Input image size after preprocessing is set as 256x256x192. The crop size based on our network is 128x128x96 with overlapping stride 24 in the test result. The batch size is set to 2, and the learning rate was set as 3e-5, as far as the initial work tested [19], swin transformer can hardly converge in the first 20-30 epochs. In the training process, we set the training epoch as 750. The default optimizer with momentum 0.9 and weight decay 2e-3 was used for model backpropagation. We employ precision, dice loss, and sensitivity three indexes to evaluate the results.

### Experiments

In this subsection, we compare the proposed model with other state-of-art methods on 3DIRCAD dataset work. CNN-based methods including UNet [6], VNet [22], Huang et al. [12] which is U-net’s optimized variant, and also ResUnet [33]. Besides, the improved graph cuts method proposed by sangsefidi et al. which is a practical new improvement for the traditional method has good performance in liver vessel segmentation [27]. In addition, there are some methods applied to data refinement [12] or specific data augmentation strategies like filters [15], note that our work does not compare to these traditional methods.

**Table 2:** Qualitative comparison of segmentation performance by three evaluation metrics on 3DIRCADb

Method	BD($\%$)	TD($\%$	Precision($\%$)	Sensitive($\%$)	Dice($\%$))
FCN	76.1	47.6	80.6${\pm }$15.3	73.8${\pm }$14.2	63.1${\pm }$15.5
VNet [22]	78.6	60.4	87.6${\pm }$11.8	75.8${\pm }$8.4	65.5${\pm }$15.4
UNETR [11]	79.7	74.1	86.1${\pm }$16.7	70.3${\pm }$6.6	66.3${\pm }$11.6
Huang et al. [12]	80.1	66.1	97.1${\pm }$0.8	74.3${\pm }$10.6	67.5${\pm }$6.9
ResUnet [33]	83.5	69.6	92.6${\pm }$1.4	71.9${\pm }$7.2	70.6${\pm }$8.5
Graph cuts (Sangse et al.) [27]	None	None	74.1${\pm }$12	None	None
IBIMHAV-Net	**85.8**	**73.6**	**98.8**${\pm }$**0.3**	**78.1**${\pm }$**2.4**	**74.8**${\pm }$**9.5**

**Fig. 7:**
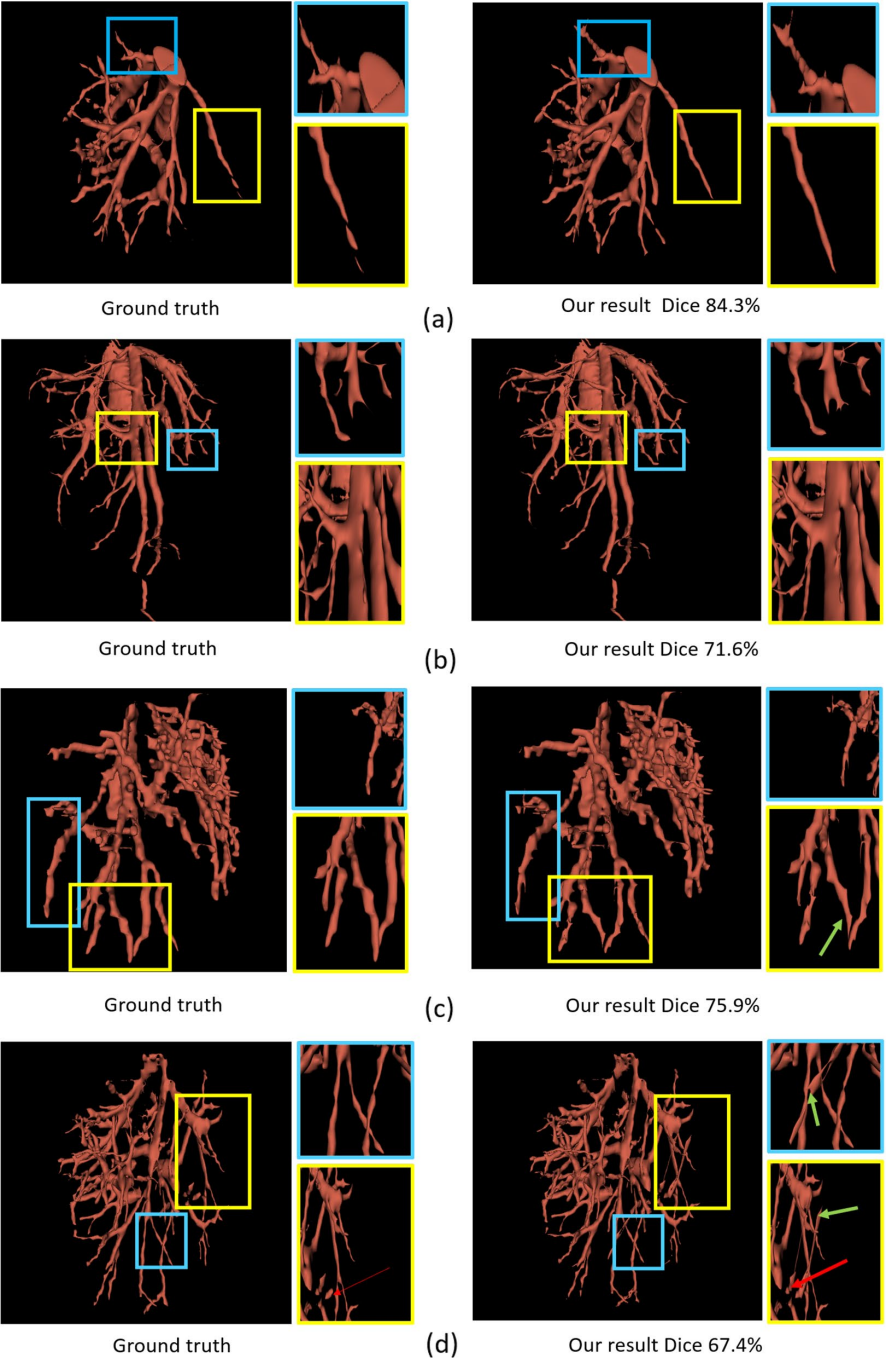
The first column list ground truth in different cases. The second column list our network’s results (a),(b),(c),(d) represent different cases

**Quantitative Results** To compare with other state-of-art methods in an equitable way, we only focus on original volume 3DIRCADb dataset. Our results are reported in Table 2. From Table 2, we can see the numerical results on two types of indexes. In order to quantize the global/local feature segmentation. we introduce two indexes which are based on centerline measurements [17] and frequently appeared in airway segmentation tasks [25]. The local/global segmentation can be measured by Branches Detected(BD)/Tree-length Detected(TD) on swin transformer’s shifted window and IB-MSA mechanism. Our model adopts larger input to catch global relationships and to obtain better segmentation results. Indeed, the CNN-based methods performed well on BD prediction which satisfies our expectations. Unetr and our model have the ability to capture global and local features so they get better TD results. However, these two indexes have higher variance than we initially expected which can only be measured by average without interval.

For the other three indexes which measure voxel results, our method exceeds other methods significantly in Dice and precision. The weighted loss function balanced segmented classes which avoid single voxel obtaining multiple labels, which leads to the larger sensitive index and prevents the over-segmentation.

Moreover, in order to achieve higher precision, our structure costs much more time and storage than the pure CNN-based methods. Here is a comparison of different models for this trade-off.

**Visualization Results**

Figure 6 shows the visualization of our experiment in one complex sample. After 3D morphological close operation and post-processing, the surface of the vessels becomes smoother and some noise blocks are removed. To compare the results visually, we utilize the software 3D slicer’s toolbox and the zoomed-in patches. The full results are shown below in Fig. 6. This sample is long and curvy, the segmentation results of FCN and 3D U-Net,3D v-Net on hepatic veins are not so well, in which some regions are over-segmented or some minor vessels are missed. The reason could be Convolutional operators limit the capability of learning long-range dependencies. In addition, the third row’s Huang et al. and ResUnet did fairly well in the whole vessel structure, yet have many errors in the vessel edge which can be seen in the zoom-in views. We can see the middle position here actually appear a fracture, it may cause by wrong labeling and it is not a small vessel so it cannot be removed by preprocessing. In our model, our design’s global feature may recognize it as cracked. Moreover, the small vessel’s segmentation at the bottom of the blue box has better completeness than UNETR. By utilizing the inductive biased multi-head attention and transformer, our methods on vessels performed relatively closer to the ground truth in vessel edges and overall structure.

To validate the generalization of our method, we conduct 4 test cases with hard cases and simple cases to show the result in Table 2. The dice coefficient in these 4 cases is 84.3, 71.6, 75.9, 67.4 respectively in Fig. 7. We can see In complex cases (c) and (d), the green arrows point to some misclassification voxels. They are caused by missing labels in the ground truth. The red arrow points to the discontinuous vessel net. It is caused by a tumor in that position.

### Ablation studies

To explore the influence of our design on the model performance, we conducted a series of ablation studies on 3Dircadb dataset.

**Influences of inductive biased positional embedding and IB-MSA** Table 3 shows the comparison of different position embedding approaches for our network. IBIMHAV-Net with general position relative bias yields 2.5% accuracy improvement compared to absolute position embedding, indicating the effectiveness of relative position bias. In addition, our proposed biased attention yields a result better than other positional embedding approaches.

**Table 3:** Inductive position bias

Position embedding methods	Precision
Our absolute + relative embedding	95.2
Relative bias embedding	97.7
Our relative bias	98.8

**Influences of more skip and transformer blocks(bottleneck)**

**Fig. 8:**
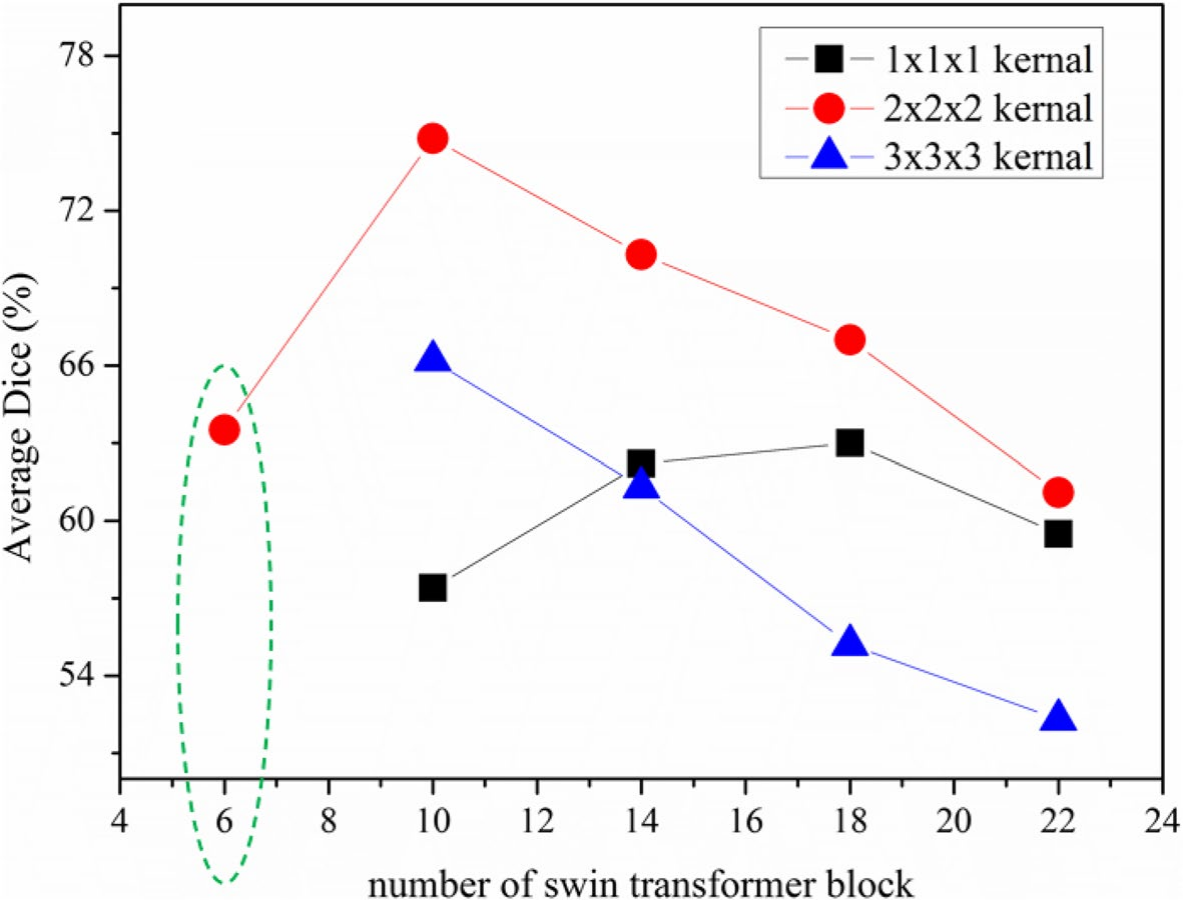
The different settings to study the effect of kernel size and model bottleneck

In our network architecture, the skip connections are connected after the down-sampling block and before the up-sampling block to unify the feature dimensions. Because the transformer has a different convergence rule compared to CNNs, which needs more discussion [1, 30]. In our model, there are only two successive Swin-transformer blocks are used to learn deep feature representation. Our experiment set 6, 10, 14, 18, 22 transformer blocks and corresponding upsampling/downsampling layers to study the convergence pattern of this model, which are shown in Fig. 8. It is worth noting that when the number of transformer blocks is 6, the smaller and larger up/down-sampling kernel blocks cannot lead to convergence.

**Effect of downsampling strategies** Patch merging is the down-sampling strategy used in the original swin transformer and the main idea is to concatenate the neighboring patches [1, 19]. We expand it to 3D by concatenating 2x2x2 neighboring patches first. Then applying a linear layer on the features can downsampling to 2x the original dimension. We choose small kernel convolution layers to reach the same operation and have better results. The results are shown in Table 4

**Table 4:** Ablation study on the impact of down-sampling DSC($\%$)

Down-sampling method	DSC for vessel	DSC for background
3D patch merging	69.12	89.95
convolution with small stride	74.83	96.92

**Effect of up-sampling strategies** The original swin transformer chooses the patch expanding layer in the encoder based on resize [1], which relies on resizing the patches to upsampling the features. We design a small kernel transposed convolution layer in the decoder to perform up-sampling as the feature dimension increases. To explore the proposed strategy’s effectiveness, we conduct the experiments of IBIMHAV-Net with Trilinear interpolation, 3D transposed convolution, and patch expanding layer([19]) on IRCADb-01 dataset. The experimental results are shown in Table 5 indicate that the proposed model combined with the transposed convolution layer can obtain better segmentation accuracy.

**Table 5:** Ablation study on the impact of upsampling

Up-sampling method	DSC for vessel	DSC for background
patch expanding	67.11	92.34
trilinear interpolation	72.21	95.22
3D transposed convolution	74.83	96.92

**Effect of local feature extraction block** The local feature extraction block includes some large kernel convolution layers. We have tried adding other feature extraction residual blocks in deeper places. The experiment shows that the CNNs can only perform well in high-resolution parts. The main reason may be that the CNNs do not have enough spatial invariant properties, which can supplement precise local features for another swin-transformer path. When we dropped this design, the accuracy, and Dice coefficient were reduced by 12% and 7.5%, respectively.

**Rolling of cropping patch size** The testing results of the proposed IBIMHAV-Net with 224 x 224 x 96, 128 x 128 x 96 input resolutions as input are presented in Table 3. As the input size increases from 224x224x96 to and the patch size remains the same as 2, the input token sequence of transformer will become larger, thus improving the segmentation performance of the model. However, although the segmentation accuracy of the model has been slightly improved $\pm 0.3\%$ DSC, the computational load of the whole network has also increased significantly. In order to balance the running efficiency of the algorithm, the experiments in this paper are based on 128x128x96 resolution scale as the input.

**Effect of the weighted loss function and post-processing** The testing results of the proposed IBIMHAV-Net structure have been discussed above. Here we design this ablation study to evaluate the necessity of these two processes. From Table 6, we know that the weighted loss function affects more than post-processing.

**Table 6:** Ablation on postprocessing and weighted loss function

Process	Postprocessing or not	Weighted Loss or Loss	Ave. Dice
Combo 1	Yes	L	71.1
Combo 2	No	Weighted Loss	73.7
Combo 3	Yes	Weighted Loss	74.8

## Conclusions

This paper designs a liver vessels segmentation method from CT images using the transformer-based network. Swin transformer has been expanding to 3D as the backbone which interleaved with convolutions and expanding for 3D volumes. In specific, the small stride convolution in both local feature block path and up/down-sampling blocks keep the spatial information hierarchically for two successive swin transformer blocks. A new pixel-wised embedding method has been used for our few samples task with variant structures. A new type of bias positional embedding in our transformer is proposed. Numerical Evaluation and visualization based on different benchmarks proved the validity of this deep learning method. Our method has been trained and tested on 3D-IRCADb-01 dataset. In the future, we would further improve segmentation accuracy by introducing more precise datasets and trying multi-task methods to reduce the negative effects of liver tumors.

## Data Availability

In this research, we utilized the 3D-IRCADb-01 dataset and resampled it to ROI. The raw data can be downloaded from(https://www.ircad.fr/research/data-sets/liver-segmentation-3d-ircadb-01/) and follow the 2.1 to resample the data.
